# Supplementary material for: Adjustment Criteria for Air-Quality Standards by Altitude: A Scoping Review with Regulatory Overview
Source: Int J Environ Res Public Health. 2025 Jun 30;22(7):1053. doi: 10.3390/ijerph22071053 (PMC12295438; doi:10.3390/ijerph22071053)
Supplement: Supplementary file 1 [file ijerph-22-01053-s001.zip › ijerph-3649864-supplementary.pdf]

Supplementary material

Table S1. Search strategy conducted for each database.

No.	**Database:** PubMed. Search conducted November 26, 2024, at 6:00 pm (Peru time	Records
#1	("Air Quality"[Title/Abstract] OR "Air Quality Standards"[Title/Abstract] OR "Air Quality Criteria"[Title/Abstract] OR "Environmental Standards"[Title/Abstract] OR "Air Quality Index"[Title/Abstract] OR "Air Quality Control"[Title/Abstract] OR "National ambient air quality standards" [Title/Abstract] OR NAAQS [Title/Abstract] OR AQI [Title/Abstract] OR "National Air Quality Index" [Title/Abstract] OR NAQI [Title/Abstract] OR "Daily Air Quality Index" [Title/Abstract] OR DAQI [Title/Abstract] OR "Comprehensive Air-quality Index" [Title/Abstract] OR CAI [Title/Abstract] OR "Pollutant Standards Index" [Title/Abstract] OR "Air quality categories" [Title/Abstract] OR AQC [Title/Abstract])	26951
#2	("Air Pollutants"[MeSH Terms] OR "Particulate Matter"[MeSH Terms] OR "Carbon Dioxide"[MeSH Terms] OR "Ozone"[MeSH Terms] OR "PM2.5"[Title/Abstract] OR "PM10"[Title/Abstract] OR "PM"[Title/Abstract] OR "NO2"[Title/Abstract] OR "SO2"[Title/Abstract] OR "O3"[Title/Abstract] OR "CO2"[Title/Abstract] OR "CO"[Title/Abstract] OR "Gaseous Pollutants"[Title/Abstract] OR "Nitrogen Dioxide"[MeSH Terms] OR "Chemical Pollutants"[Title/Abstract] OR "Pollutants Transport"[Title/Abstract])	1,189,451
#3	("Altitude"[MeSH Terms] OR "Altitude"[Title/Abstract] OR "Highlands"[Title/Abstract] OR "High Altitude"[Title/Abstract] OR "Elevated Altitude"[Title/Abstract] OR Mountain [Title/Abstract] OR "Mountain Areas"[Title/Abstract] OR "Great altitude" [Title/Abstract] OR "3500 m" [Title/Abstract] OR "3500 m.a.s.l."[Title/Abstract] OR "3500 m a.s.l." [Title/Abstract] OR "3,500 m a.s.l." [Title/Abstract])	62,116
#4	#1 AND #2 AND #3	189

No.	Datab**ase:** SCOPUS. Search conducted November 26, 2024, at 6:00 pm (Peru time)	Records
#1	(TITLE-ABS-KEY ("Air Quality" OR "Air Quality Standards" OR "Air Quality Criteria" OR "Environmental Standards" OR "Air Quality Index" OR "Air Quality Control" OR "National ambient air quality standards" OR "NAAQS" OR "AQI" OR "National Air Quality Index" OR "NAQI" OR "Daily Air Quality Index" OR "DAQI" OR	149,343

	"Comprehensive Air-quality Index" OR "CAI" OR "Pollutant Standards Index" OR "Air quality categories" OR "AQC"))	
#2	(TITLE-ABS-KEY ("Air Pollutants" OR "Particulate Matter" OR "Carbon Dioxide" OR "Ozone" OR "PM2.5" OR "PM10" OR "PM" OR "NO2" OR "SO2" OR "O3" OR "CO2" OR "CO" OR "Gaseous Pollutants" OR "Nitrogen Dioxide" OR "Chemical Pollutants" OR "Pollutants Transport"))	4,024,418
#3	(TITLE-ABS-KEY ("Altitude" OR "Highlands" OR "High Altitude" OR "Elevated Altitude" OR "Mountain" OR "Mountain Areas" OR "Great altitude" OR "3500 m" OR "3500 m a s l" OR "3 500 m a s l"))	474,826
#4	#1 AND #2 AND #3	**1,801**

No.	**Database:** Web of Science. Search conducted November 26, 2024, at 6:00 pm (Peru time)	Records
#1	TS=("Air Quality" OR "Air Quality Standards" OR "Air Quality Criteria" OR "Environmental Standards" OR "Air Quality Index" OR "Air Quality Control" OR "National ambient air quality standards" OR "NAAQS" OR "AQI" OR "National Air Quality Index" OR "NAQI" OR "Daily Air Quality Index" OR "DAQI" OR "Comprehensive Air-quality Index" OR "CAI" OR "Pollutant Standards Index" OR "Air quality categories" OR "AQC")	79,047
#2	TS=("Air Pollutants" OR "Particulate Matter" OR "Carbon Dioxide" OR "Ozone" OR "PM2.5" OR "PM10" OR "PM" OR "NO2" OR "SO2" OR "O3" OR "CO2" OR "CO" OR "Gaseous Pollutants" OR "Nitrogen Dioxide" OR "Chemical Pollutants" OR "Pollutants Transport")	2,729,233
#3	TS=("Altitude" OR "Highlands" OR "High Altitude" OR "Elevated Altitude" OR "Mountain" OR "Mountain Areas" OR "Great altitude" OR "3500 m" OR "3500 m a s l" OR "3 500 m a s l")	230,541
#4	#1 AND #2 AND #3	941

No.	**Database:** Gale OneFile: Environmental Studies and Policy. Search conducted November 26, 2024, at 6:00 pm (Peru time).	Records
#1	"Air Quality" OR "Air Quality Standards" OR "Air Quality Criteria" OR "Environmental Standards" OR "Air Quality Index" OR "Air Quality Control" OR "National Ambient Air Quality Standards" OR "NAAQS" OR "AQI" OR "Pollutant Standards Index" OR "Ambient Air Standards"	3,918
#2	"Air Pollutants" OR "Particulate Matter" OR "Carbon Dioxide" OR "Ozone" OR "PM2.5" OR "PM10" OR "NO2" OR "SO2" OR "O3" OR "CO2" OR "CO" OR "Gaseous Pollutants" OR "Nitrogen Dioxide" OR "Chemical Pollutants" OR "Air Pollution"	45,320
#3	"Altitude" OR "High Altitude" OR "Highlands" OR "Elevated Altitude" OR "Mountain" OR "Mountain Areas" OR "Great Altitude" OR "High Elevation" OR "Elevated Areas" OR "2500 m"	11,462
#4	#1 AND #2 AND #3	43

Table S2. Reasons for exclusion of documents during full-text review (n=6).

Title	Year	Journal	Authors	Decision
1. Changes in particulate matter concentrations at different altitudinal levels with environmental dynamics	2015	Journal of Animal & Plant Sciences,	Z. Zona, Z. Ali, S. Sidra, A. Nimra, M. Ahmad, K. Aziz, I. Zainab, Quratulain, B. Ansari, S. T. Raza, Z. A.Nasir, I. Colbeck	They monitored ambient PM concentrations at different locations and altitudes but did not establish, adjust, or apply corrections for AQS
2. Vertical profiles of NO3, N2O5, O3, and NOx in the nocturnal boundary layer: 2. Model studies on the altitude dependence of composition and chemistry	2024	Journal of Geophysical Research,	Andreas Geyer, Jochen Stutz	The study identified nighttime pollutant concentration profiles and their variation with altitude. The model depicts vertical transport and fluxes but does not apply any adjustments
3. Spatial sensitivities of human health risk to intercontinental and high-altitude pollution	2013	Atmospheric Environment	Jamin Koo, Qiqi Wang, Daven K. Henze, Ian A. Waitz, Steven R.H. Barrett	The study assess human Sensitivities to pollutants relevant to intercontinental and high-altitude PM pollution are calculated
4. Comparison of air quality at different altitudes from multi-platform measurements in Beijing	2018	Atmospheric, Chemistry and Physics	Hongzhu Ji, Siying Chen, Yinchao Zhang, He Chen, Pan Guo, and Peitao Zhao	The study assesses human sensitivities to pollutants relevant to intercontinental and high-altitude PM pollution but does not establish any AQS adjustments

5. A meteorological analysis of PM10 episodes at a high altitude city and a low altitude city in central Greece - The impact of wood burning heating devices	2018	Atmospheric Research	Konstantinos Dimitriou, Pavlos Kassomenos	The study analyzed daily PM_{10} concentrations in two cities in central Greece: Karpenisi, a high-altitude city, and Lamia, a low-altitude city. However, no AQS adjustment analysis was conducted.
6. A practical computerized atmospheric pollution dispersion model with altitude-dependent diffusivities: application to carbon monoxide concentration calculation	2007	International Journal of Environmental Studies	D. C. Chou, J.Y. Sung	The study focuses on altitude-dependent turbulent diffusion coefficients and their effect on pollutant concentration distribution. However, no adjustment calculations were performed.
